# Human Liver Cell Trafficking Mutants: Characterization and Whole Exome Sequencing

**DOI:** 10.1371/journal.pone.0087043

**Published:** 2014-01-23

**Authors:** Fei Yuan, Erik L. Snapp, Phyllis M. Novikoff, Sylvia O. Suadicani, David C. Spray, Barry Potvin, Allan W. Wolkoff, Pamela Stanley

**Affiliations:** 1 Marion Bessin Liver Research Center, Albert Einstein College of Medicine, New York, New York, United States of America; 2 Department of Cell Biology, Albert Einstein College of Medicine, New York, New York, United States of America; 3 Department of Anatomy and Structural Biology, Albert Einstein College of Medicine, New York, New York, United States of America; 4 Department of Pathology, Albert Einstein College of Medicine, New York, New York, United States of America; 5 Department of Neuroscience, Albert Einstein College of Medicine, New York, New York, United States of America; 6 Department of Medicine, Albert Einstein College of Medicine, New York, New York, United States of America; 7 Department of Urology, Albert Einstein College of Medicine, New York, New York, United States of America; Emory University School of Medicine, United States of America

## Abstract

The HuH7 liver cell mutant *Trf1* is defective in membrane trafficking and is complemented by the casein kinase 2α subunit CK2α’’. Here we identify characteristic morphologies, trafficking and mutational changes in six additional HuH7 mutants *Trf2-Trf7. Trf1* cells were previously shown to be severely defective in gap junction functions. Using a Lucifer yellow transfer assay, remarkable attenuation of gap junction communication was revealed in each of the mutants *Trf2-Trf7*. Electron microscopy and light microscopy of thiamine pyrophosphatase showed that several mutants exhibited fragmented Golgi apparatus cisternae compared to parental HuH7 cells. Intracellular trafficking was investigated using assays of transferrin endocytosis and recycling and VSV G secretion. Surface binding of transferrin was reduced in all six *Trf2-Trf7* mutants, which generally correlated with the degree of reduced expression of the transferrin receptor at the cell surface. The mutants displayed the same transferrin influx rates as HuH7, and for efflux rate, only *Trf6* differed, having a slower transferrin efflux rate than HuH7. The kinetics of VSV G transport along the exocytic pathway were altered in *Trf2* and *Trf5* mutants. Genetic changes unique to particular *Trf* mutants were identified by exome sequencing, and one was investigated in depth. The novel mutation Ile34Phe in the GTPase RAB22A was identified in *Trf4*. RNA interference knockdown of RAB22A or overexpression of RAB22AI34F in HuH7 cells caused phenotypic changes characteristic of the *Trf4* mutant. In addition, the Ile34Phe mutation reduced both guanine nucleotide binding and hydrolysis activities of RAB22A. Thus, the RAB22A Ile34Phe mutation appears to contribute to the *Trf4* mutant phenotype.

## Introduction

Membrane trafficking is an essential process responsible for maintaining the structure, composition and functions of eukaryotic cells [1]. There are two major membrane trafficking routes, endocytic and exocytic, that govern regulated transport between the plasma membrane, Golgi apparatus, endoplasmic reticulum (ER), endosomes and lysosomes [2]. The endocytic pathway is used for the internalization of macromolecules such as signaling receptors from the plasma membrane. Internalized molecules are sorted to early endosomes and, either directed to late endosomes and subsequently to lysosomes for degradation, or recycled back to the cell surface directly, or via recycling endosomes [3]–[5]. The exocytic pathway, on the other hand delivers newly synthesized proteins from the ER, through the Golgi apparatus to the plasma membrane [6]. Each step of membrane trafficking - cargo selection, vesicle formation, vesicle movement along cytoskeletal elements, tethering and fusion with target membrane - is stringently controlled [7]. Of key importance is the superfamily of RAB GTPases that ensure efficient transport of cargo to the appropriate destination [2], [7], [8].

In order to investigate diverse intracellular trafficking pathways and their regulation in liver cells, we developed a dual selection strategy to isolate trafficking mutants from the human hepatocarcinoma cell line HuH7 [9]. The ligands ASOR (asialo-orosomucoid) and ovalbumin, that bind distinct membrane receptors, were conjugated with a toxin and allowed to internalize into HuH7 cells via receptor-mediated endocytosis. The first mutant isolated for dual resistance to both ligands was *Trf1*. *Trf1* cells exhibit altered trafficking of the asialoglycoprotein receptor (ASGPR), increased sensitivity to Pseudomonas exotoxin A (PEx), and defective gap junction assembly and functions [9], [10]. Complementation expression cloning identified the casein kinase 2α subunit CK2α’’ as a potential basis for the *Trf1* phenotype, which was largely corrected by overexpression of a cDNA encoding CK2α’’ [11], [12]. Further studies showed that phosphorylation of the ASGPR cytoplasmic domain by CK2α’’ is required for association of several chaperones, which might explain the redistribution of ASGPR in *Trf1* cells [13]. Subsequently, we isolated six additional *Trf* mutants, *Trf2-Trf7*, with properties related to, but distinct from, *Trf1* [14].

In this paper we show that, like *Trf1* [10], *Trf2-Trf7* mutants are also defective in dye transfer via gap junctions, that many have an altered Golgi apparatus morphology, and some are affected in endocytic or exocytic membrane trafficking pathways. Efforts to identify the molecular basis of *Trf2-Trf7* mutations using next-generation exome sequencing revealed several candidate mutations, one of which, a novel Ile34Phe mutation in RAB22A, appears to be partly responsible for the *Trf4* phenotype.

## Results

### Defective Gap Junction Communication in *Trf* Mutants

**Figure 1:**
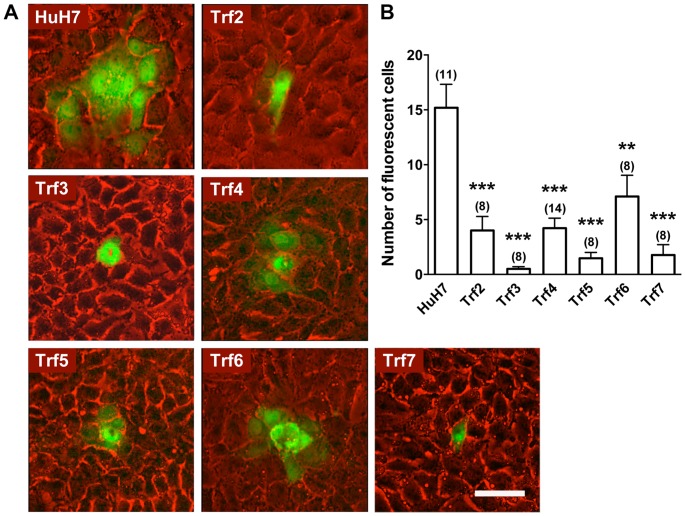
Dye transfer assay. (A) Lucifer yellow was microinjected into single cells in a confluent monolayer and dye spread to neighboring cells after 3 min (shown as green) was visualized using fluorescence microscopy. Scale bar 50 µm. (B) The extent of dye transfer was quantified by counting the number of fluorescent cells around each injected cell. Data are expressed as mean ± SEM. Numbers above each bar indicate the number of cells injected. Significance was determined by Student’s t-test. **p<0.01, ***p<0.001. Data were obtained in two experiments with HuH7 cells included in each experiment giving, respectively, 17±2.8 (n = 8) and 11±1.5 (n = 3) cells with dye. These numbers are not significantly different (*p* = 0.24).

Functional gap junctions are often determined by examining the efficiency of fluorescent dye spreading from cell to cell in monolayer culture [15]. The *Trf1* mutant was previously shown to be severely defective in the transfer of Lucifer yellow [10], and this was subsequently shown to be corrected by overexpression of CK2α” (unpublished observations). To investigate *Trf2-Trf7* mutants, Lucifer yellow was microinjected into single cells of each mutant, and after three min, images were acquired. As shown in Fig. 1A, transfer of Lucifer yellow to adjacent cells was substantial in HuH7 cells within three min, showing that gap junction channels were functional. In contrast, the efficiency of dye spreading in each of the six *Trf* mutants was markedly reduced, with few neighboring cells showing dye coupling (Fig. 1A). The lowest degree of dye coupling was manifested in the *Trf3* mutant (Fig. 1B). These results demonstrate that gap junction-mediated intercellular communication is defective in each *Trf* mutant.

### Morphologic Changes in HuH7 Trafficking Mutants

**Figure 2:**
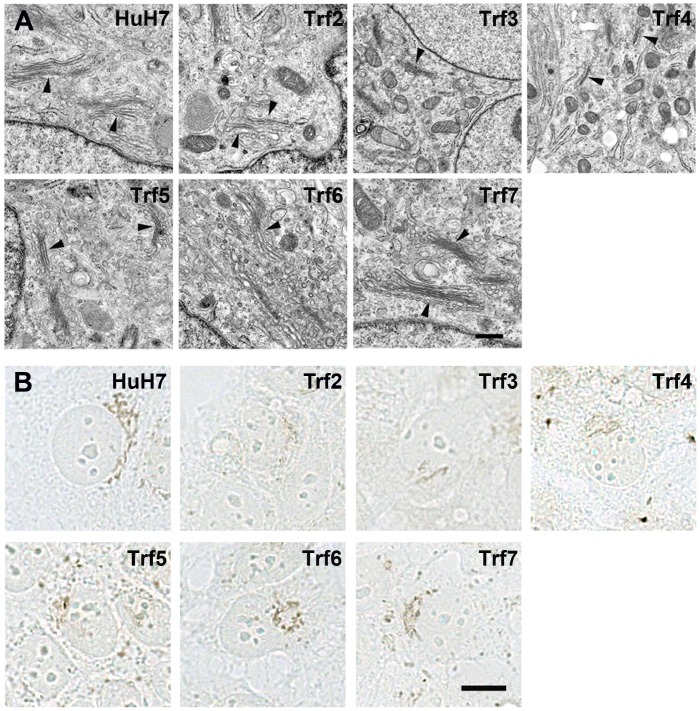
The Golgi apparatus in *Trf* mutants. (A) Representative EM images showing perinuclear Golgi apparatus membrane stacks (arrowhead) in HuH7 and *Trf* mutant cells. Scale bar: 0.5 µm. (B) Representative histochemical staining of the Golgi apparatus lumen with TPPase substrate. Scale bar: 10 µm.

The Golgi apparatus is a hub of membrane trafficking. We examined the Golgi apparatus in 15–20 cells of each *Trf* mutant by electron microscopy. Cells that were sectioned at the center of the cell and contained both a Golgi apparatus and a centriole were chosen for analysis. The Golgi apparatus in HuH7 cells was an extensive and continuous series of membranes, with a characteristic stacked structure containing 4–5 cisternae located at the periphery of the nucleus (Fig. 2A). In contrast, the Golgi apparatus in *Trf2* cells appeared as a complex of membranes composed of discontinuous or fragmented stacked cisternae compared to those in HuH7 cells. *Trf3, Trf4* and *Trf5* displayed fragmented Golgi membranes resembling the Golgi apparatus of *Trf2* cells. However, the Golgi organization in *Trf6* and *Trf7* cells appeared to be more similar to HuH7 cells. Morphometric analysis of single sections revealed that only the Golgi apparatus in *Trf7* cells was significantly different from HuH7 cells in length, whereas *Trf4*, *Trf6* and *Trf7* Golgi apparati were different from HuH7 cells in width (Fig. S1). Note that fragmentation of Golgi membranes was not reflected in the morphometric analysis on single sections.

Golgi apparatus structure was also examined by light microscopy by staining for the well-established Golgi apparatus marker enzyme thiamine pyrophosphatase (TPPase) using a substrate that deposits a brown-black reaction product within Golgi cisternae [16]. In HuH7 cells, the Golgi apparatus appeared as an extensive continuous membranous network adjacent to the nucleus (Fig. 2B). In *Trf2* cells, the Golgi apparatus appeared as multiple dot-like structures and short linear stretches, consistent with the results from electron microscopy showing discontinuous membrane stacks. Similarly fragmented Golgi cisternae were present in *Trf3*, *Trf4*, *Trf5* and *Trf6* cells. The Golgi apparatus in *Trf7* cells was similar to HuH7 cells, although exhibiting a less extensive network near the nucleus. Fragmentation of the Golgi apparatus is typical of cells in mitosis [17]. However, methyl-green pryronin staining showed that mitotic figures were present in only a few percent of cells in a monolayer, as would be expected, whereas the 15–20 cells examined for each *Trf* mutant had the Golgi apparatus morphology typical of that mutant.

### Endocytosis and Efflux of Transferrin in *Trf* Mutants

Previously we reported that the HuH7 *Trf* mutants exhibit altered endocytosis of fluorescent ASOR, a well-characterized ligand that undergoes endocytosis and traffics to lysosomes [14]. To determine whether endocytic recycling is also affected, we investigated transferrin (Tfn) endocytosis and efflux in the *Trf* mutants.

**Figure 3:**
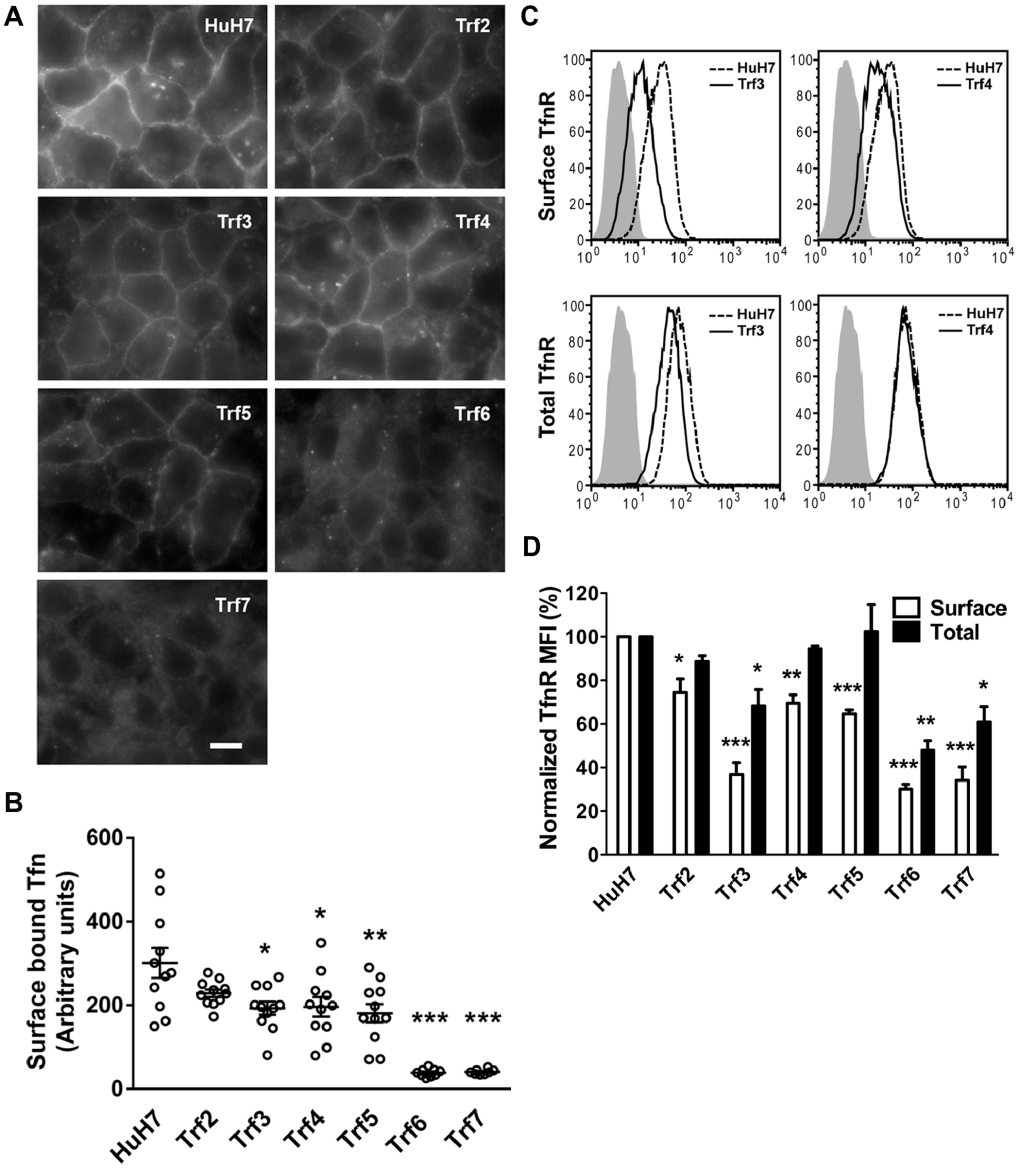
Surface Tfn binding and TfnR distribution. (A) Cells were washed and incubated with Alexa Fluor 488-labeled Tfn on ice for 30 min prior to fixation. Fixed cells were imaged using a widefield microscope. Scale bar: 10 µm. (B) The mean fluorescence of surface Tfn was measured by NIH ImageJ software and is presented as mean ± SEM (n = 11). The data are representative of two independent experiments. (C) Total and cell surface TfnR expression were analyzed by flow cytometry of fixed cells incubated with either FITC-conjugated anti-TfnR antibody or isotype control, with or without cell permeabilization. Total and surface TfnR profiles for *Trf3* and *Trf4* are shown with a solid line and HuH7 TfnR profiles are shown with a dashed line. The data are representative of three experiments. (D) The histogram represents cell surface or total TfnR normalized to HuH7 cells and are expressed as mean ± SEM, n = 3. Statistical analysis was performed using a Student’s t-test. *p<0.05, **p<0.01, ***p<0.001.

We first assessed the binding of Tfn to the cell surface. Parental and *Trf* mutant cells were incubated with fluorescein-conjugated Tfn on ice for 30 min. After three washes to remove unbound Tfn, cells were fixed for imaging. HuH7 cells showed robust labeling of fluorescent Tfn at the plasma membrane. However, less surface-bound Tfn was observed in each of the six mutants (Fig. 3A). In *Trf6* and *Trf7* cells, surface staining was almost too weak to identify cell boundaries. Based on quantification of mean fluorescence index (MFI) at the surface of ∼10 cells, *Trf3*, *Trf4* and *Trf5* bound 40–45% less Tfn than HuH7 cells and very little surface binding of Tfn was evident in *Trf6* or *Trf7* cells (Fig. 3B). The extent of the reductions observed varied somewhat from our previous report using an I^125^-Tfn binding assay [14]. This may reflect differences between the two types of assays.

To determine the steady state distribution of Tfn receptors (TfnR) in the *Trf* mutants, we performed flow cytometry analysis on fixed versus permeabilized cells using a fluorescein-conjugated anti-TfnR antibody. Typical profiles comparing surface TfnR (from fixed cells) and total TfnR (from permeabilized cells) in HuH7 cells compared to *Trf3* and *Trf4* mutant cells are shown in Fig. 3C. It can be seen that HuH7 cells express slightly more surface TfnR than *Trf4* and significantly more than *Trf3* cells. Normalized MFI data show that total TfnR levels were similar in HuH7 and *Trf2, Trf4* and *Trf5* mutants, whereas total TfnR levels were reduced by ∼30% in *Trf3* and by ∼50–60% in *Trf6* and *Trf7* cells (Fig. 3D). Surface detection of TfnR was reduced in all *Trf* mutants, with the largest reductions exhibited by *Trf3, Trf5, Trf6* and *Trf7* mutants (Fig. 3D). Therefore, TfnRs were redistributed in the *Trf* mutants, and the levels of surface TfnR correlated approximately with relative Tfn binding (Fig. 3B).

**Figure 4:**
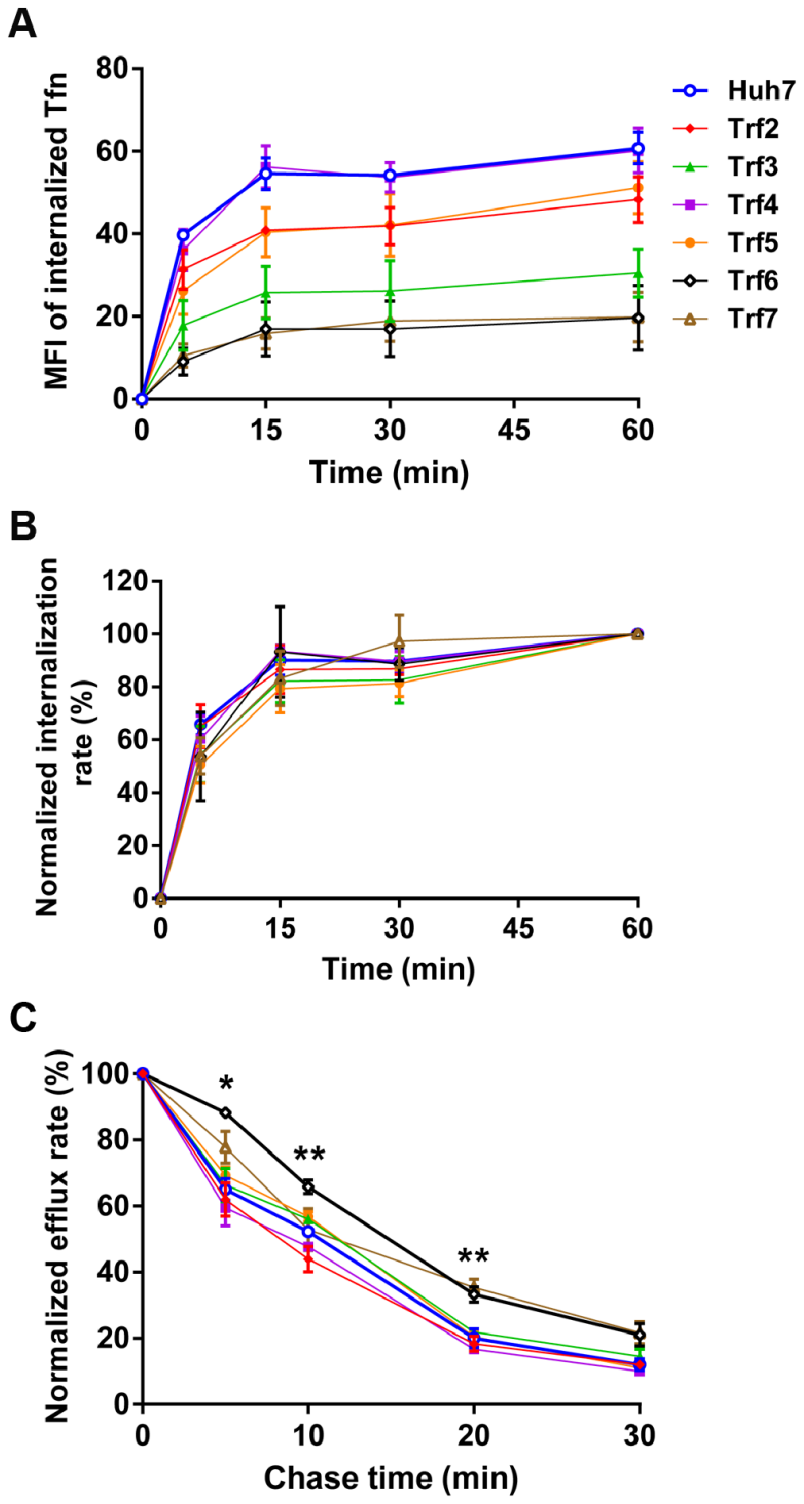
Transferrin internalization and efflux. (A) After serum starvation, cells were treated with or without 10 µg/ml Alexa Fluor 488-labeled Tfn at 37°C for up to 60 min. Following acid washing to remove surface-bound Tfn, internalized Tfn was determined by flow cytometry. Data are expressed as mean ± SEM of three independent experiments. (B) The internalization rate was calculated using MFI at each time point normalized to MFI at 60 min for each mutant and presented as mean ± SEM. (C) Alexa Fluor 488-labeled Tfn was allowed to internalize at 37°C for 20 min prior to chase for the indicated times at 37°C in the presence of 0.1 mM desferroxamine and excess unlabeled Tfn. The amount of fluorescent Tfn remaining in cells was determined by flow cytometry. The efflux rate was normalized to 0 min efflux (100%) in each cell line, and is represented as mean ± SEM of three independent experiments. Significance was determined using a Student’s t-test. *p<0.05, **p<0.01.

To investigate Tfn endocytosis in the *Trf* mutants, the rate of Tfn internalization under steady state conditions was determined. Fluorescent Tfn was allowed to internalize at 37°C for up to 60 min prior to acid washing to remove surface-associated Tfn. The MFI of internalized Tfn measured by flow cytometry reached a plateau after ∼15 min uptake in all cell lines. Internalized Tfn in *Trf4* and *Trf5* at 60 min was similar to that in HuH7 cells, but the other *Trf* mutants had a significantly reduced amount of Tfn, with *Trf6* and *Trf7* having the least, consistent with their low numbers of surface TfnR (Fig. 4A). However, when the curves were normalized to the amount of internalized Tfn at 60 min, as an indicator of endocytosis rate, all *Trf* mutants were comparable to HuH7 cells (Fig. 4B).

We also investigated Tfn efflux kinetics in the *Trf* mutants. Upon Tfn uptake for 20 min at 37°C, internalization was stopped and unbound Tfn was removed by the addition of the iron chelator desferroxamine and excess, unlabeled Tfn. Incubation was continued at 37°C for the indicated “chase” times, in the presence of desferroxamine and unlabeled Tfn. Fluorescent Tfn that remained in the cells was determined by flow cytometry and MFIs were normalized to 0 min efflux to calculate the rate of efflux. In all mutants except *Trf6*, the Tfn efflux rate was similar to that in HuH7 parental cells. However, *Trf6* exhibited a significantly delayed efflux rate at several time points (Fig. 4C).

### Secretory Pathway Kinetics are Perturbed in *Trf2* and *Trf5* Mutants

**Figure 5:**
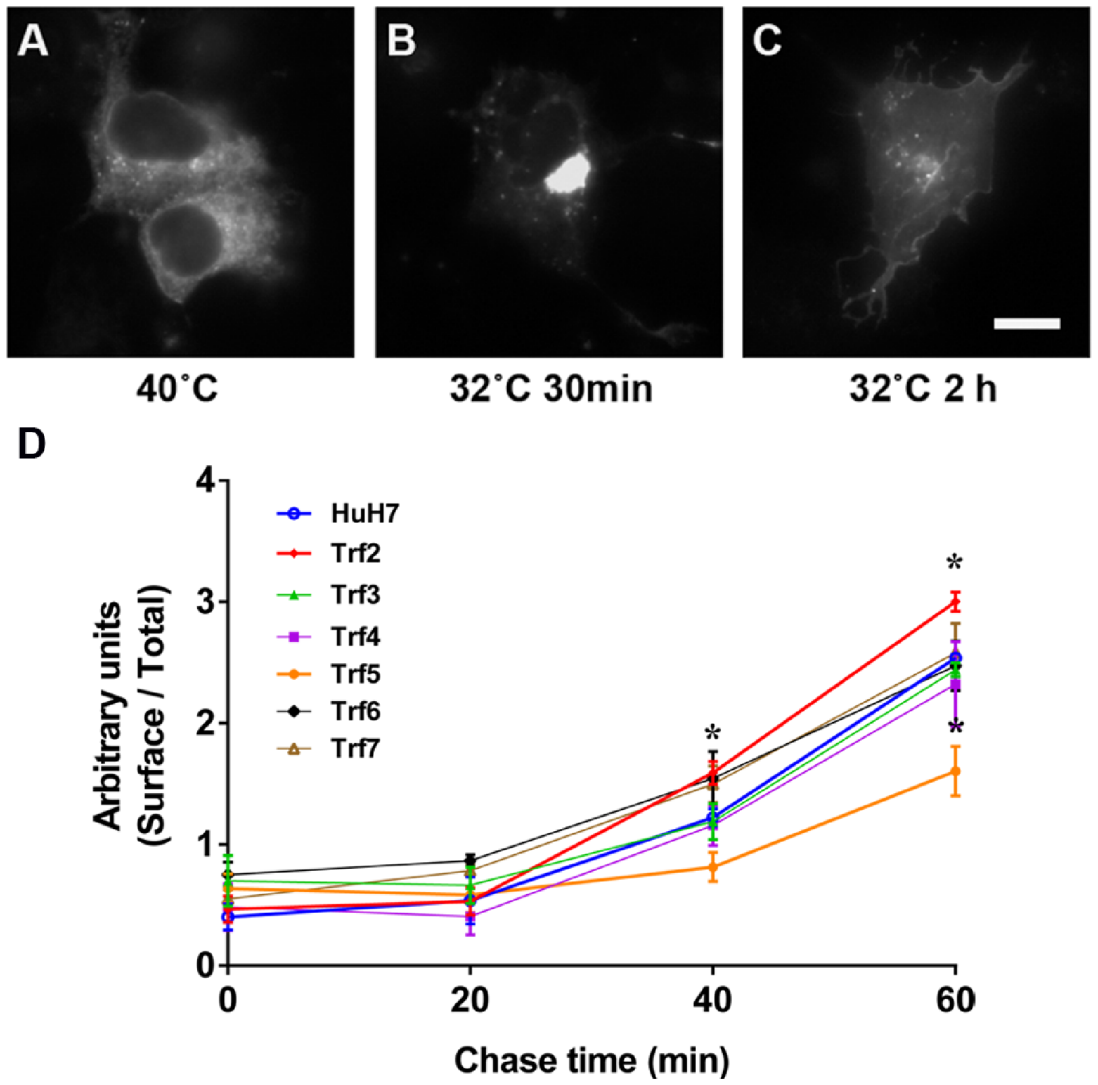
VSV G transport from ER to the cell surface. (A–C) Cells transfected with plasmids encoding VSV G-EGFP were incubated at 40°C overnight. The cells were then shifted to 32°C in medium containing cycloheximide for various times, fixed and imaged by fluorescence microscopy to detect VSV G-EGFP. Scale bar: 10 µm. (D) Surface VSV G-EGFP was determined using I1-hybridoma supernatant containing an antibody recognizing the extracellular domain of VSV G [35], and normalized against total VSV G-EGFP expression quantitated by flow cytometry analysis. Results are shown as mean ± SEM of three experiments. Significance was determined by Student’s t-test. *p<0.05.

To determine whether ER-to-plasma membrane trafficking is affected in the *Trf* mutants, we monitored transit of the G glycoprotein of Vesicular Stomatitis Virus (VSV). The temperature-sensitive mutant *ts045* VSV G tagged at the C-terminus with EGFP (VSV G-EGFP) is a representative cargo protein often used to follow constitutive secretion. This mutant VSV G misfolds and is retained in the ER at 40°C. Shifting to the permissive temperature of 32°C allows it to fold correctly, and begin transport from the ER via Golgi apparatus compartments to the plasma membrane. VSV G-EGFP transport in HuH7 cells was visualized by fluorescence microscopy. At 40°C, VSV G-EGFP displayed typical ER distribution (Fig. 5A). Upon shifting to 32°C for 30 min, VSV G-EGFP was found accumulating in the perinuclear region, in the location of the Golgi apparatus (Fig. 5B). Two hours after incubation at 32°C, VSV G-EGFP reached the plasma membrane (Fig. 5C). We then determined the time course of VSV G appearance at the plasma membrane versus total VSV G expression after temperature shift by flow cytometry, in the presence of cycloheximide to inhibit new protein synthesis. As shown in Fig. 5D, delivery of VSV G-EGFP to the cell surface was delayed in *Trf5* cells, while in *Trf2* cells the transit of VSV G-EGFP was slightly faster compared to HuH7 cells. The remaining four *Trf* mutants exhibited similar kinetics to parental HuH7 cells.

### Whole Exome Sequencing of *Trf* Mutants

In attempts to identify a mutation in each *Trf* mutant that might provide the basis for their respective pleiotropic phenotypes, whole exome sequencing was performed on genomic DNA from HuH7 and the five mutants *Trf2*, *Trf3*, *Trf4*, *Trf5* and *Trf7*. Only 6 samples could be analyzed per lane and thus *Trf6* was not included. We anticipated that a subset of genetic variations in the exomes of the highly related HuH7 lines should reveal useful candidates for the ultimate identification of the genetic origin of each *Trf* mutant.

Genomic DNA samples were whole exome captured and sequenced using Illumina HiSeq 2000 technology, as described in Methods. For each sample, ∼30 million reads were aligned to the human reference genome (NCBI build 37/hg19), with ∼75% of targeted bases achieving 10-fold or greater coverage. The average read depth ranged from 23 to 31 nucleotides in targeted regions. After quality filtering, a total of 40,913 SNPs were retained, including known SNPs documented in the public database dbSNP build 132, and novel SNPs. We selected SNPs that caused non-synonymous nucleotide substitutions in an exon. We then identified SNPs unique to each mutant, i. e. SNPs that were absent from parental HuH7 genomic DNA, and from the other four *Trf* mutant exomes (Table 1). Similarly, we analyzed small insertions and deletions (Indels), and found about 6,200 per mutant after filtering. Indels unique to each mutant that caused “frame shift” or “codon changes” were identified.

**Table 1:** SNPs and Indels detected in exomes of HuH7 and *Trf* mutants.

Source genomicDNA	HuH7	*Trf2*	*Trf3*	*Trf4*	*Trf5*	*Trf7*
**Filtered SNPs**	33133	31673	31407	31471	31329	31409
**SNPs in** **dbSNP 132**	32145	30492	30370	30441	30317	30412
**Novel SNPs**	988	1181	1037	1030	1012	997
**Non-synonymous SNPs**	6492	6195	6115	6156	6099	6111
**Unique non-synonymous SNPs**	**1824**	**97**	**28**	**78**	**28**	**22**
**Filtered** **Indels**	6287	6269	6172	6246	6215	6202
**Non-synonymous Indels**	348	363	348	353	357	343
**Unique non-synonymous Indels**	**59**	**7**	**3**	**7**	**8**	**3**

Candidate genes that might provide the basis of a *Trf* mutant phenotype were selected based on the following criteria: 1) the encoded protein contained relevant motif(s) such as a phosphoinositide binding domain (e. g. FYVE domain, PX or PH domain) which have been implicated in playing a role in membrane trafficking [18]; 2) the SNP was in a functional motif of the candidate protein; 3) the SNP was in a conserved site; 4) the protein was reported to be involved in intracellular trafficking according to the literature. Using these factors in combination, top candidates were selected for further validation. Five heterozygous mutations from four *Trf* mutants were confirmed by Sanger sequencing (Table 2). Among them, the mutation in the small GTPase RAB22A seemed a promising candidate for contribution to the *Trf4* mutant phenotype.

**Table 2:** Missense variants validated by sequencing.

Gene(Mutant)	Description	Genotype	mRNA	aa change	Prediction algorithms	
					SIFT^1^	Polyphen-2^2^
VPS13B (*Trf2*)	Vacuolar protein sorting-associated protein 13B	Het	8983T>C	S2995P	0.00 (D)	0.999 (D)
RAB22A (*Trf4*)	Ras-related protein RAB22A	Het	100A>T	I34F	0.04 (D)	1.00 (D)
WDFY3 (*Trf5*)	WD repeat and FYVE-containing protein 3	Het	3241G>C	G1081R	0.37 (T)	0.242 (B)
EGFR (*Trf5*)	Epidermal growth factor receptor	Het	125G>T	G42V	0.03 (D)	0.999 (D)
CKAP5 (*Trf7*)	Cytoskeleton- associated protein 5	Het	1330C>A	L444I	0.23 (T)	0.999 (D)

1 SIFT score ranges from 0 to 1. The amino acid substitution is predicted “damaging (D)” if the score is < = 0.05, and “tolerated (T)” if the score is >0.05.

2 Polyphen-2 score ranges from 0 to 1. The amino acid substitution is predicted “probably damaging (D)” if the score is between 0.957 and 1; “possibly damaging (P)” if the score is between 0.453 and 0.956; “benign (B)” if it is between 0 and 0.452.

### *Trf4* has a Functional Mutation in RAB22A

RAB22A is a Ras GTPase superfamily member, which encodes a 194 aa protein. Whole exome sequencing revealed a missense change on chromosome 20: c.100A>T (p. Ile34Phe) on one allele of the *RAB22A* gene as a novel polymorphism unique to *Trf4*. The algorithms SIFT and Polyphen-2 were used to predict functional effects of the Ile34Phe change, and both predicted this mutation as likely to be deleterious to the activity of RAB22A (Table 2). Sanger sequencing confirmed the presence of the Ile34Phe change in genomic DNA and in cDNA from *Trf4*.

**Figure 6:**
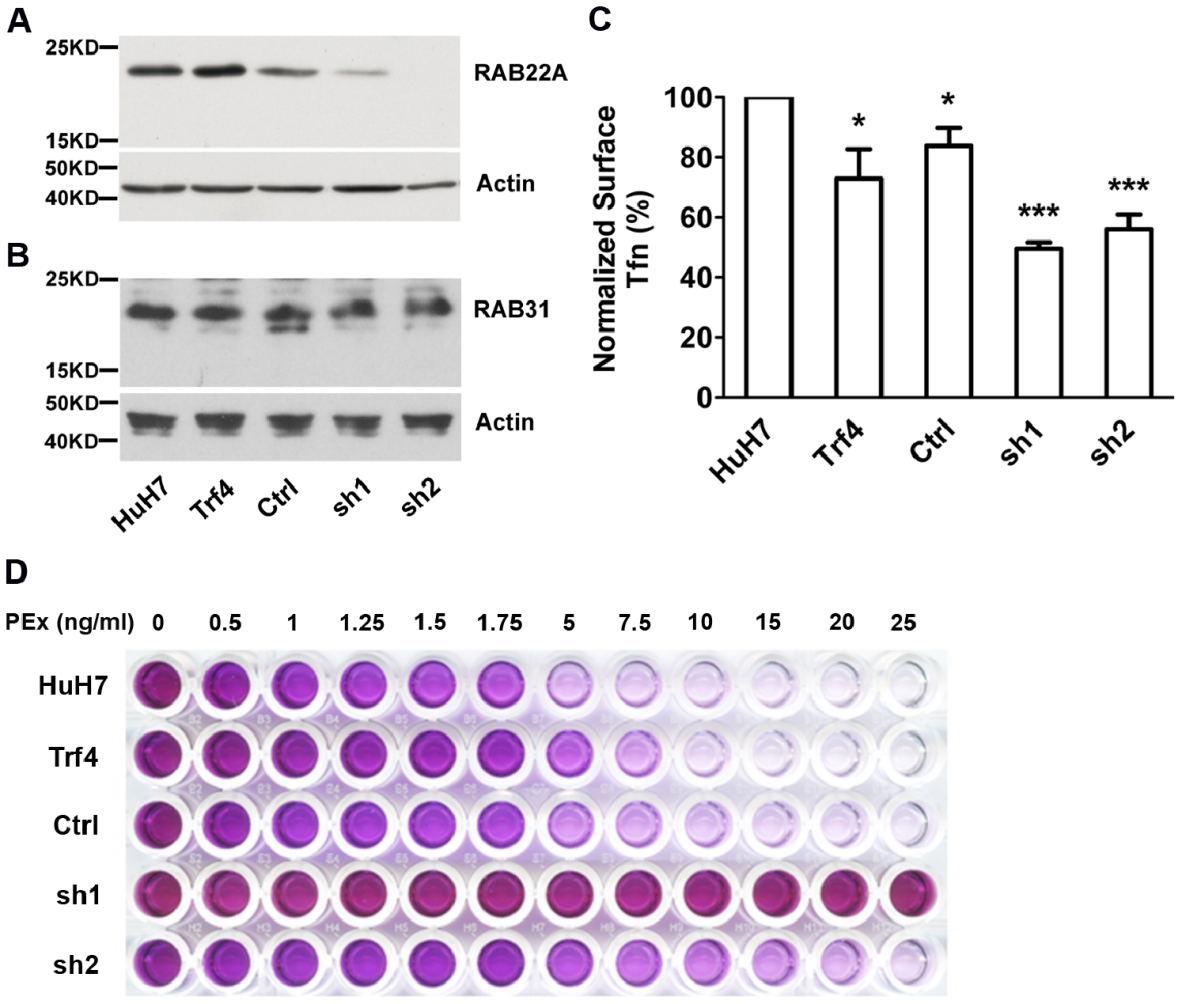
Depletion of RAB22A in HuH7 cells. (A) Western blot showing endogenous RAB22A in HuH7 and *Trf4*, as well as in HuH7 expressing empty vector (Ctrl) or RAB22A shRNAs (sh1 and sh2), as detected with anti-RAB22A antibody. Actin was a loading control. The data are representative of blots from three independent lysates of stable HuH7 sh1 and sh2 populations. The ratio of RAB22A to actin bands determined by NIH ImageJ was not significantly different for HuH7 versus HuH7 vector or *Trf4* but RAB22A was significantly reduced in sh1 (*p*<0.04) and sh2 (*p*<0.0001). (B) The independent lysates from (A) were examined with anti-RAB31 antibody. There was no significant difference RAB31 expression between HuH7, *Trf4* and HuH7 cells expressing sh1 or sh2. (C) Surface Tfn binding was analyzed after incubation of Alexa Fluor 488-labeled Tfn with cells on ice for 30 min followed by flow cytometry. Results were normalized to surface Tfn in HuH7 cells and are shown as mean ± SEM, n = 4. (D) MTT assay of cells incubated with different concentrations of PEx at 37°C for 4 days. Cells were treated with MTT solution at 37°C for 3 hours. Cell viability was reflected by the color of the formazan crystals solubilized in DMSO. The data are representative of two independent experiments. Statistical significance was determined by Student’s t-test. *p<0.05, ***p<0.001.

RAB22A has been reported to regulate recycling of cargo proteins such as MHC-I and Tfn in Hela cells [19], [20]. To identify roles for RAB22A in HuH7 cells, we investigated loss-of-function effects of endogenous RAB22A knockdown. Two shRNA sequences (sh1 and sh2) that targeted RAB22A mRNA in the coding region were delivered into HuH7 cells using lentiviral vectors, and stable cell populations were selected based on resistance to puromycin. A control cell line was also generated using lentiviral empty vector. Western blot analysis showed that *Trf4* expressed a comparable amount of endogenous RAB22A to HuH7 cells, whereas RAB22A was dramatically reduced in both knockdown cell lines, especially in sh2 (Fig. 6A). Levels of RAB22B, also known as RAB31, were not reduced by sh1 or sh2 (Fig. 6B).

One feature that could readily differentiate *Trf4* from HuH7 cells is Tfn surface binding (Fig. 3A, 3B). To assess the RAB22A knockdown lines for Tfn binding, they were incubated with fluorescently labeled Tfn on ice for 30 min and, after washing, analyzed by flow cytometry. Similar to *Trf4,* both RAB22A knockdown cell lines exhibited decreased surface Tfn binding (Fig. 6C), although Tfn binding to sh1- and sh2-expressing HuH7 cells was not statistically different (*p* = 0.28). Another phenotype examined was PEx resistance. *Trf4* was about 2-fold more resistant than parental HuH7 cells, as reported previously [14]. The two RAB22A knockdown lines showed much greater resistance to PEx than *Trf4* cells (Fig. 6D). Although sh2 reduced RAB22A to a greater extent than sh1, this was not reflected in their relative abilities to reduce Tfn binding or induce PEx resistance. Nevertheless, the combined results show that HuH7 cells with very low levels of RAB22A exhibited *Trf4*-like properties.

### RAB22AI34F Exhibits Reduced GTP Binding and Hydrolysis

**Figure 7:**
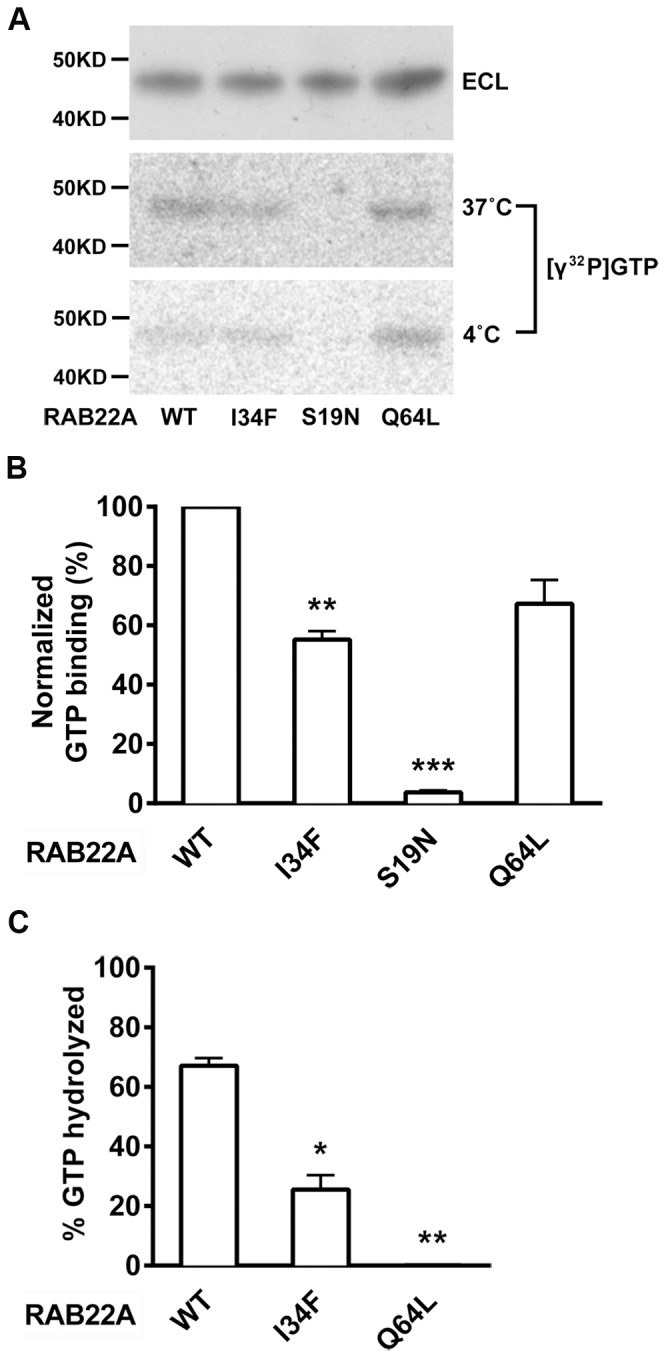
GTP binding and GTPase activity of sfGFP-RAB22AI34F. sfGFP-tagged RAB22A and mutant proteins expressed by HEK-293T cells were captured from lysate on anti-GFP agarose, eluted and resolved by SDS-PAGE, and the blot transferred to NC membranes. GTP overlay was performed to determine GTP binding and hydrolysis as described in Materials and Methods. (A) Western blot analysis was performed using anti-GFP antibody. The two replicate blots were incubated with [γ^ 32^P]GTP for one hour at RT then incubated in hydrolysis buffer containing Mg^2+^ either at 4°C or at 37°C for 2 hours. (B) GTP binding was normalized to the binding of sfGFP-RAB22A WT. (C) GTP hydrolysis was quantified as the percent reduction in radioactivity after incubation at 37°C relative to GTP bound at 4°C (100%). Results are shown as mean ± range of two independent experiments. Significance was determined by Student’s t-test. *p<0.05, **p<0.01, ***p<0.001.

RAB GTPases cycle between GDP-bound (inactive) and GTP-bound (active) conformations. Active GTP-bound forms are associated with membranes and responsible for recruiting specific downstream effectors to target subcellular compartments [8]. Therefore, *in vitro* GTP binding and hydrolysis assays have been extensively used to evaluate RAB activity. There are two mutations in RAB22A that have been well characterized [21]. The Ser19Asn RAB22A mutation is a dominant negative mutation that binds little GTP. In contrast, substitution of Gln by Leu to give Q64L generates a constitutively active RAB22A, defective in GTP hydrolysis. To evaluate GTP binding and hydrolysis activity of the Ile34Phe RAB22A mutation found in *Trf4*, plasmids encoding superfolder GFP (sfGFP) [22] linked to the N-terminus of RAB22A or RAB22A with each mutation I34F, S19N or Q64L were expressed in HEK-293T cells, and the recombinant proteins were purified from cell lysates on anti-GFP beads. Binding of radioactive GTP was determined using a modified GTP overlay assay. GTP binding to sfGFP-RAB22AI34F was significantly reduced compared to binding of wild type sfGFP-RAB22A (Fig. 7A, 7B). As expected, GTP binding to sfGFP-RAB22AS19N was almost not detectable (Fig. 7A, 7B). A GTP hydrolysis assay showed that sfGFP-RAB22AQ64L hydrolyzed less than 1% of bound GTP, while sfGFP-RAB22AI34F hydrolyzed ∼25% after 2 hours of incubation at 37°C (Fig. 7A, 7C). In contrast, wild type sfGFP-RAB22A hydrolyzed ∼67% of bound GTP. These results show that sfGFP-RAB22AI34F had reduced binding of GTP, and its ability to hydrolyze GTP was also defective.

### RAB22AI34F Overexpression Results in Enlarged Vesicles

**Figure 8:**
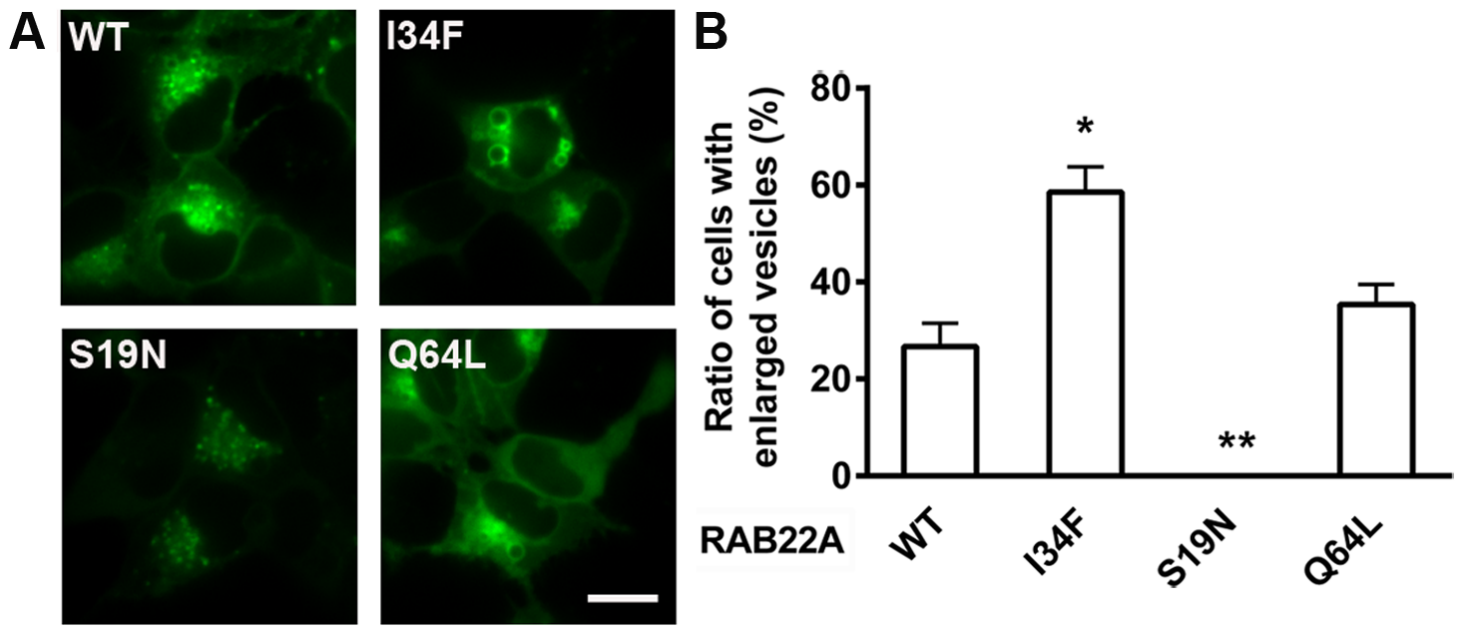
Enlarged vesicular structures in sfGFP-RAB22AI34F expressed in HEK-293 cells. Live HEK-293 cells were imaged using spinning-disk confocal microscope 48 hours after transfection with various sfGFP-RAB22A constructs as shown (A). Scale bar: 10 µm. From 61-372 cells were examined for enlarged spherical structures and the percentage is expressed as mean ± SEM of three independent experiments (B). Significance was determined by Student’s t-test. *p<0.05, **p<0.01.

Interestingly, we observed prominent vesicular enlargement in HEK-293 cells transfected with sfGFP-RAB22A, but not in overexpressing HuH7 cells. Analogous phenomena have been reported previously when wild type or constitutively active RAB proteins, including RAB22A, are overexpressed in certain cell lines [19], [20]. In our experiments, 26.7±4.8% sfGFP-RAB22A and 35.4±4.1% sfGFP-RAB22AQ64L expressing cells contained at least one enlarged vesicle, whereas 58.6±5.2% sfGFP-RAB22AI34F expressing cells were found to be positive for similar vesicles (Fig. 8A, 8B). Using confocal microscopy, these vesicles were further shown to be spherical and mobile (Fig. S2). Moreover, the average number of fluorescent spheres in sfGFP-RAB22AI34F expressing cells was greater than those in the wild type sfGFP-RAB22A and sfGFP-RAB22AQ64L expressing cells. In contrast, sfGFP-RAB22AS19N localized in punctate, mainly perinuclear, vesicles.

### RAB22AI34F is a Novel RAB22A Mutant

The properties of RAB22AI34F represent a combination of those exhibited by the dominant negative RAB22AS19N mutation and the constitutively active RAB22AQ64L mutation. To determine if RAB22AI34F could induce the *Trf4* phenotype, cloned HuH7 transfectants sorted for expression of sfGFP-RAB22AI34F using the GFP tag were compared for Tfn binding by flow cytometry. Four HuH7 clones expressing sfGFP-RAB22AI34F exhibited reduction in Tfn binding compared to HuH7 cells (23%, 44%, 54% and 87% as much Tfn, respectively). Similar results were obtained with three clones of HuH7 cells expressing sfGFP-RAB22AQ64L that bound 49%, 50% and 58% Tfn compared to HuH7. Interestingly, *Trf4* transfectant clones sorted for expression of sfGFP-RAB22A had Tfn binding similar to that of *Trf4,* ∼40% compared to HuH7 cells. Therefore, overexpression of sfGFP-RAB22A did not rescue the *Trf4* phenotype, suggesting that other factors may contribute to this phenotype, or that RAB22AI34F behaves as a dominant negative mutant.

## Discussion

In this paper, we compare gap junction function, Golgi apparatus morphology and common trafficking pathways in six *Trf* mutants derived from HuH7 cells by following ligands and cargo through endocytic recycling and secretory pathways. Combined with earlier studies on ASGPR trafficking [14], both degradative and recycling pathways were compromised in all six mutants, while the secretory pathway for VSV G was affected in *Trf2* and *Trf5* mutants. In addition, severely reduced gap junction communication observed in each *Trf* mutant may reflect defects in the trafficking of gap junction channel proteins. Since several kinds of trafficking routes were altered, and in most mutants, Golgi apparatus structure was also altered, the mutants represent valuable tools to identify genes essential for general trafficking through multiple intracellular compartments.

Expression cloning is often used to isolate the gene responsible for a specific phenotype. We applied this strategy to the characterization of the *Trf1* mutant and cloned CK2α’’ which complements many features of the *Trf1* phenotype [11], [12]. However, expression cloning is very laborious, so we sought an alternative approach to identify mutations in the other *Trf* mutants. Recently, whole exome sequencing has proven to be considerably useful for the identification of causative alleles underlying not only human diseases [23], but also certain traits [24]. By comparing exome sequences from unrelated or closely related samples, novel and harmful variations shared by a group of affected individuals have been successfully discovered [23], [25], [26]. In our case, to uncover molecular bases of trafficking defects, we sequenced the exomes of parental HuH7 and five *Trf* mutants. After analysis of unique genetic variations for each mutant that distinguished it from the other five “relatives”, a novel mutation in RAB22A, Ile34Phe, was identified as a potential candidate for the basis of the *Trf4* phenotype. Other validated candidates (Table 2) await detailed investigation.

RAB22A colocalizes with the early endosomal markers Rab5 and EEA1 and physically interacts with EEA1 [19], [27], consistent with a role in early endocytosis. Functionally, RAB22A regulates recycling of membrane proteins endocytosed via both clathrin-mediated and clathrin-independent pathways, represented by transferrin receptor and MHC-1 respectively [19], [20]. Given that TfnR expression at the plasma membrane was affected in *Trf4*, we postulated that RAB22AI34F might contribute to the *Trf4* phenotype.

In RAB22A, Ile34 is fully conserved in all species examined, including zebrafish, frog, chicken and mammals. Alignment analysis among 20 human RABs indicated that Ile at this position is only present in RAB22A, and the closely related RAB22B/RAB31. Conversely, Phe is observed at the corresponding position in RAB3A, RAB8A and RAB27A [28]. Importantly, Ile34 is located in the switch I region (aa 29–42), 3 aa upstream of a motif TIGXXF (where X is variable), that is highly conserved throughout the RAB family [29], [30]. As one of the two critical switch regions in RABs, switch I undergoes a conformational change during GTP/GDP conversion, and subsequently affects recruitment of downstream effectors [31]. Previous studies have already revealed aa residues in the switch I region that are functionally significant, such as Lys82 in RAB34 [32] and Gly43 in RAB27A [28]. Mutations at these positions “inactivate” RABs by abolishing the association of specific effectors.

Based on the GTP binding and hydrolysis properties of the RAB22AI34F mutant, together with its ability to induce enlarged cytoplasmic vesicles, it appears that RAB22AI34F combines aspects of the properties of RAB22AS19N and RAB22AQ64L. Depletion of RAB22A in HuH7 cells using shRNAs, or overexpression of RAB22AI34F, led to a *Trf4*-like phenotype. This suggests that the RAB22AI34F mutation acts dominantly since *Trf4* is heterozygous for the RAB22AI34F mutation. A dominant effect may also explain why introduction of RAB22A into *Trf4* cells did not rescue the *Trf4* phenotype. This experiment is complicated by the fact that overexpression of wild type RAB22A may result in phenotypes similar to knockdown, as reported previously [19], [20]. Nevertheless, the combined data identify Ile34 as an important aa for RAB22A function, and suggest that the RAB22AI34F mutation contributes in part to the *Trf4* mutant phenotype.

## Materials and Methods

### Cells

*Trf* mutants were previously isolated from the human hepatocarcinoma cell line HuH7 using a dual selection protocol as described [14]. HEK-293 (CRL-1573) and HEK293T (CRL-3216) cells were obtained from the American Type Culture Collection. Cells were grown in RPMI 1640 medium or Dulbecco’s modified Eagle’s medium supplemented with 10% fetal bovine serum (FBS), 100 U/ml penicillin and 100 µg/ml streptomycin. Confluent, or near confluent, cells were used for each experiment.

### Gap Junction Assay

Lucifer yellow (LY) microinjection was used to evaluate the degree of gap junction communication, as previously described [33]. For LY microinjections, single HuH7 cells or mutants in confluent cultures were impaled with a glass microelectrode containing LY (5% by weight in 150 mM LiCl). LY iontophoresis was performed by applying 0.1 µA current for 3 min using an electrometer (Model 3100; A-M Systems, WA). LY fluorescence images were acquired immediately after removal of the microelectrode from the injected cells. Quantification of the degree of dye coupling was performed by counting the number of cells to which LY spread from the injected cell.

### Electron Microscopy (EM) and Light Microscopy (LM)

Cells were washed in serum free medium before fixation in an aldehyde fixative (mixture of 4% paraformaldehyde and 2.5% glutaraldehyde in 0.1 M cacodylate buffer, pH 7.4) for 10 min, and rinsed in 7.5% sucrose [16]. For EM studies, cells were post-fixed in 1% osmium tetroxide, dehydrated in a series of ethanol dilutions from 70% to 100%, and rinsed with propylene oxide followed by embedding in Epon. The procedures were carried out with cells attached to glass coverslips. Ultrathin sections (200–300 angstroms) were prepared using an LKB Ultramicrotome (Leica Microsystems, Deerfield, IL), stained in lead citrate and imaged in the electron microscope (Philips 300 and JEOL instrument in the Analytical Imaging Facility). The EM studies focused on structures of the perinuclear region, specifically the stacks of cisternae of the Golgi apparatus. For LM studies, fixed samples were incubated with buffers containing thiamine pyrophostate, the substrate of a marker enzyme thiamine pyrophosphatase (TPPase) as previously described [16].

### Surface Binding of Transferring

Cells were grown in 8-well Lab-tek chambers (Thermo Scientific, Rochester, NY) until confluent. After washing with PBS, cells were serum starved in serum free medium (SFM) at 37°C for 1 hour. The cells were then chilled on ice and incubated with 20 µg/ml Alexa Fluor 488-conjugated Tfn (T-13342, Life Technologies, Grand Island, NY) in SFM on ice for 30 min. Excess Tfn was removed by washing and cells were fixed with 4% PFA at room temperature (RT) for 15 min. Images of the fixed cells were acquired in bright field and FITC channel using a 60×1.4 NA oil objective on an Olympus 1×71 inverted microscope equipped with a Lambda DG-4 excitation source and Lambda 10-2 emission filter wheel (Sutter Instrument, Novato, CA). The mean fluorescence intensity of surface Tfn was measured using ImageJ (http://rsbweb.nih.gov/ij/).

### Surface and Total Transferrin Receptor Expression

Cells were removed from dishes after 5 min of treatment with enzyme-free cell dissociation solution (S-014, Millipore, Billerica, MA). Dissociated cells were either fixed with 4% PFA (for surface TfnR) or fixed and permeabilized using 0.1% Triton X-100 (for total TfnR). Subsequently cells were incubated with FITC-conjugated anti-TfnR antibody (M-A712, BD Biosciences, San Jose, CA) on ice for 30 min, or with FITC-conjugated mouse anti-human IgG2a (sc-2856, Santa Cruz Biotechnology, Inc., Santa Cruz, CA) under the same conditions as control staining. After two washes with ice-cold PBS, cells were processed for surface and total TfnR quantitation by flow cytometry using a BD FACScan (Becton, Dickinson and Company, New Jersey, NJ). Flow cytometry results were analyzed using FlowJo software (Tree Star Inc., Ashland, OR). The mean fluorescence index (MFI) of control IgG labeled cells was subtracted from each sample.

### Transferrin Internalization and Efflux Assays

Cells were grown on a 6-well plate until confluent. For the Tfn internalization assay, after PBS washing, cells were serum starved for one hour in SFM, and then incubated with 10 µg/ml Alexa Fluor 488-conjugated Tfn in SFM at 37°C for up to 60 min. Control cells were treated in the same way but with medium free of Tfn. Tfn internalization was stopped by putting the cells on ice and washing immediately with ice-cold PBS. Surface bound Tfn was further removed by mild acid washing (15 mM citric acid, 150 mM NaCl, pH 4.6) followed by ice-cold PBS washes for three times. Cells were then detached with enzyme-free cell dissociation solution and processed for flow cytometry using BD FACScan.

For Tfn efflux, serum starved cells were incubated with prewarmed 10 µg/ml Alexa Fluor488-Tfn in SFM at 37°C for 20 min before chilling on ice to stop internalization. After three washes with ice-cold PBS, the cells were incubated with prewarmed SFM containing 0.1 mM desferroxamine (Sigma-Aldrich, St. Louis, MO) and 0.5 mg/ml unlabeled Tfn (Sigma-Aldrich) at 37°C for up to 30 min. The cells were then placed on ice, washed with ice-cold PBS, dissociated with enzyme-free cell dissociation solution and collected for flow cytometry analysis. Fluorescent Tfn remaining in cells at each time point were measured and the efflux rate was expressed as percentage of initial intracellular Tfn at 0 min of efflux.

### VSV G-GFP Trafficking from ER to the Cell Surface

HuH7 cells grown on glass-bottomed 3.5 cm MatTek dishes (MatTek Corporation, Ashland, MA) were transfected with a plasmid encoding *ts045* VSV G tagged at the N-terminus of enhanced GFP [34] using GenJet reagent (SL100489-HUH, SignaGen Laboratories, Rockville, MD). 5 hours after transfection, cells were moved to 40°C and incubated overnight to allow VSV G-EGFP accumulating at ER. Then the cells were incubated in fresh SFM containing cycloheximide (100 µg/ml) at 32°C for different times, and fixed with 4% PFA. Images were captured in bright field and FITC channel using Olympus 1×71 inverted microscope with a 60×1.4 NA oil objective and DG-4 setting.

For quantitation purposes, cells grown in 6-well plates were transfected with VSV G-EGFP expressing plasmids and cultured at 40°C overnight. The next day, cycloheximide (100 µg/ml) was added 30 min prior to shifting cells to 32°C for various times. The cells were then collected using enzyme-free cell dissociation solution and rinsed once with ice-cold blocking buffer (PBS containing 2% FBS and 0.1% NaN_3_). Surface VSV G-EGFP was labeled on ice for 30 min with mouse I1-hybridoma supernatant (CRL-2700, American Type Culture Collection, Manassas, VA) containing an antibody that specifically recognizes VSV G ectodomain [35]. After washing twice with blocking buffer, the cells were incubated with Alexa Fluor 647-conjugated anti-mouse IgG antibody (A-21235, Life Technologies) on ice for 30 min. Following final two washes with blocking buffer, the cells were analyzed by BD FACScan. MFI of ∼5000 cells surface VSV G-EGFP (Cy5 channel) was normalized against total (GFP channel) and the ratios are shown in the figure.

### Whole Exome Sequencing

Genomic DNA was prepared using the Puregene genomic DNA isolation protocol (Qiagen, Valencia, CA) from HuH7, *Trf2*, *Trf3*, *Trf4*, *Trf5* and *Trf7* cells. Trueseq Libraries were made according to Illumina’s standard Truseq DNA Prep protocol and then captured using Trueseq Capture protocol. Six samples were captured in one reaction as recommended, and the captured DNA was sequenced on the HiSeq 2000 sequencer as 100 bp paired-end reads using Trueseq sequencing reagents. Library preparation, exome enrichment and sequencing were performed by the Epigenomics Shared Facility at Albert Einstein College of Medicine. Reads were aligned to the human reference genome (hg19 assembly) using the Burrows–Wheeler Aligner (BWA) tool [36]. Alignments were merged and marked for duplication using Picard (http://picard.sourceforge.net). Local realignment, base quality score recalibration and variant calling (UnifiedGenotyper) were performed using the Genome Analysis Toolkit (GATK) v1.4–9 [37]. Variants were selected from the Illumina TruSeq capture regions, filtered and variant quality scores were recalibrated by GATK. Finally, the resulting VCF files were annotated using snpEff v2.0.5 [38]. Functional prediction of non-synonymous amino acid substitutions was performed using SIFT (http://sift.jcvi.org) [39] and Polyphen-2 (http://genetics.bwh.harvard.edu/pph2/) [40] algorithms. Sanger DNA sequencing was used to confirm the presence of mutated alleles in selected candidates.

### Western Blot Analysis

Cells cultured in 6 cm dishes were collected and lysed in 50 mM Tris-HCl, pH 8.0, 150 mM NaCl, 1% Triton X-100, 1 mM EDTA and 1% protease inhibitor cocktail (P8340, Sigma-Aldrich). Cell lysates containing equal amounts of protein determined by the BCA protein assay (Pierce Biotechnology, Rockford, IL) were loaded onto 12% SDS-PAGE gels, electrophoresed and then transferred to polyvinyldifluoride (PVDF) membrane (Perkin Elmer, Waltham, MA). After blocking in TBS/Tween buffer (50 mM Tris-HCl, 150 mM NaCl, 0.1% Tween 20, pH7.6) containing 10% nonfat milk, the membrane was probed with rabbit anti-RAB22A polyclonal antibody (12125-1-AP, Proteintech, Chicago, IL) or rabbit anti-RAB31 polyclonal antibody (16182-1-AP, Proteintech) at 4°C overnight. Horseradish peroxidase-conjugated goat anti-rabbit IgG (Santa Cruz Biotechnology, Inc.) was used as secondary antibody. Mouse anti-β-actin antibody (A5441, Sigma-Aldrich) followed by horseradish peroxidase-conjugated goat anti-mouse IgG was used as loading control. After incubation with each antibody, the membrane was washed five times for 5 min each with TBS/Tween 20. Blots were developed using the ECL plus kit (Pierce Biotechnology) according to the manufacturer’s instructions.

### Toxin Sensitivity Assay

The toxin sensitivity of cells was determined as described previously [11], [14]. In brief, cells were trypsinized and resuspended at a density of 2×10^5^ cells/ml in fresh culture medium. A cell suspension (10^4^ cells in 50 µl) was added to each well of a 96-well plate. Different concentrations of Pseudomonas exotoxin A (Sigma-Aldrich) were prepared in culture medium, and 50 µl was added per well. The cells were incubated at 37°C for 4 days when cells in the control well (without toxin) became confluent. Cells were then incubated with Thiazolyl Blue Tetrazolium Bromide (Sigma-Aldrich) at 37°C for 3 hours. After careful washing, the colored formazan crystals were dissolved in 200 µl DMSO and recorded in a Tecan Sunrise microplate reader (Tecan Group, Mannedorf, Germany). Absorbance was measured at 540 nm with background subtraction at 650 nm.

### shRNA Interference of RAB22A

The RAB22A shRNA targeting plasmids in the pLKO.1 lentiviral vector were obtained from the TRC Genome-Wide shRNA collection (shRNA1 targeted bases 94–114 of the coding region: CCAAACATCAACCCAACAATA; shRNA2 (targeted bases 453–473 of the coding region: CGCGATAAACATAAATGAACT). Plasmid DNA was transfected into HEK-293T cells together with three packaging plasmids (pMDLg/pRRE, pRSV-Rev and pMD2.G). Empty vector was used as control. The resulting supernatant was collected after 48 hours and used to infect HuH7 cells. A stable cell population was selected in the presence of 5 µg/ml puromycin. The expression level of RAB22A in knockdown cells was determined by western blot analysis using anti-RAB22A antibody. To determine knockdown specificity, expression of RAB31 (also known as RAB22B), which is highly homologous to RAB22A, was detected using anti-RAB31 antibody.

### GTP Binding and GTP Hydrolysis

The GTP binding abilities of recombinant RAB22A proteins conjugated to the C-terminus of superfolder GFP (sfGFP) was measured using a modified GTP overlay assay [21], [41]. Briefly, RAB22A cDNA coding sequences were inserted into *Bgl*II and *Sal*I sites of the sfGFP-C1 plasmid. HEK-293T cells were transfected with sfGFP-RAB22A expression plasmids using the calcium phosphate method. Forty-eight hours after transfection, cells were collected and lysed with IP buffer (50 mM Tris-HCl pH8.0, 150 mM NaCl, 1% NP-40). GFP-tagged proteins were captured with anti-GFP-Agarose (MBL International, Woburn, MA) and resolved on a 12% SDS-PAGE gel followed by transfer to nitrocellulose membranes. One membrane was probed with anti-GFP antibody (ab2430, Abcam, Cambridge, MA) and revealed by ECL as loading control. The other two membranes were incubated with binding buffer (50 mM NaH_2_PO_4_ pH7.5, 10 µM MgCl_2_, 0.2% Triton X-100, 2 mM DTT, 10 µM ATP) at room temperature for 30 min, then for another hour in fresh binding buffer containing 10 µci/ml [γ^ 32^P]GTP (Perkin Elmer, Waltham, MA). After four washes with binding buffer, membranes were incubated with hydrolysis buffer (20 mM Tris-HCl, pH 8.0, 1 mM DTT, 5 mM MgCl_2_) at either 37°C or 4°C for 2 hours. Then the membranes were washed, dried and radioactive GTP was visualized and quantitated by phosphorimaging.

### Confocal Microscopy

HEK-293 cells were grown on collagen-coated, glass-bottomed 3.5 cm MatTek dishes (MatTek, Ashland, MA) for 24 hours and transfected with sfGFP-RAB22A expression plasmids as described above. After 48 hours, images in the FITC channel were captured using a 60×1.4 NA oil immersion objective on a CARV II spinning-disk imager (Crisel Instruments, Rome, Italy) equipped with an iXon back-illuminated 897 EMCCD camera (Andor Technologies, Belfast Ireland). All images were analyzed using ImageJ software (http://rsbweb.nih.gov/ij/) and Adobe Photoshop (Adobe Systems, San Jose, CA).

### Overexpression of RAB22A and RAB22A Mutants in HuH7 or *Trf4* Cells

HuH7 or *Trf4* cells were transfected with wild type sfGFP-RAB22A or mutant sfGFP-RAB22AI34F and sfGFP-RAB22AQ64L expression plasmids as described above using GenJet reagent. After 48 hours, transfected cells were selected for resistance to 500 µg/ml G418 (Mediatech, Herndon, VA). Single clones were picked and Tfn binding was determined using flow cytometry analysis of the GFP positive population.

### Statistical Analysis

Statistical analysis was performed using the Student’s t-test calculated using Microsoft Excel. Errors were determined as standard error of the mean (SEM).

## Supporting Information

Figure S1 **Morphometric analysis of the Golgi apparatus in** ***Trf*** **mutants.** The length and width of the Golgi apparatus in 12–23 cells from EM sections of each *Trf* mutant were measured using a user defined protocol in Volocity 6.3 (PerkinElmer, Waltham MA). The results are expressed as mean ± SEM. Statistical analysis was performed by Student’s t-test. *p<0.05, ***p<0.001.
(TIF)

Figure S2 **Structure and motility of enlarged vesicles.** (A) XY, XZ, and YZ projections of enlarged vesicles in HEK-293 cells transfected with sfGFP-RAB22AI34F. (B) Confocal projection show enlarged RAB22AI34F positive vesicle locating in an intact cell. (C) Time series show mobile activities of buds on the surface of the spherical vesicles. All images were captured using a Zeiss Duoscan confocal microscope with a 63×1.4 NA oil objective, 489 nm laser, 500–550 nm bandpass filter. Scale bar: 10 µm.
(TIF)
