# Supplementary figures and images for: Human Liver Cell Trafficking Mutants: Characterization and Whole Exome Sequencing
Source: PLoS One. 2014 Jan 23;9(1):e87043. doi: 10.1371/journal.pone.0087043 (PMC3900707; doi:10.1371/journal.pone.0087043)

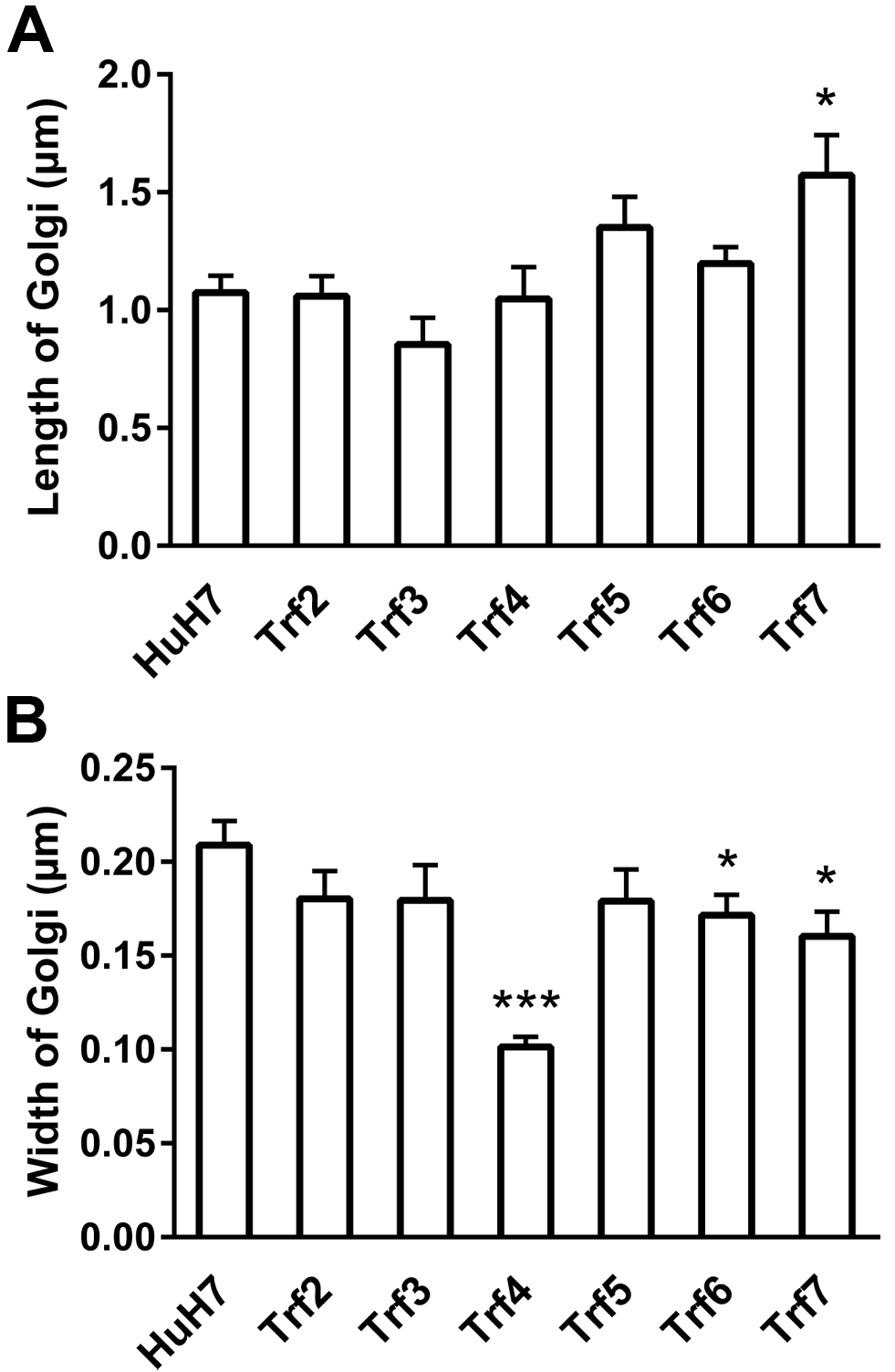

Supplement: Figure S1 — Morphometric analysis of the Golgi apparatus in Trf mutants. The length and width of the Golgi apparatus in 12–23 cells from EM sections of each Trf mutant were measured using a user defined protocol in Volocity 6.3 (PerkinElmer, Waltham MA). The results are expressed as mean ± SEM. Statistical analysis was performed by Student’s t-test. *p<0.05, ***p<0.001. (TIF) [file pone.0087043.s001.tif]

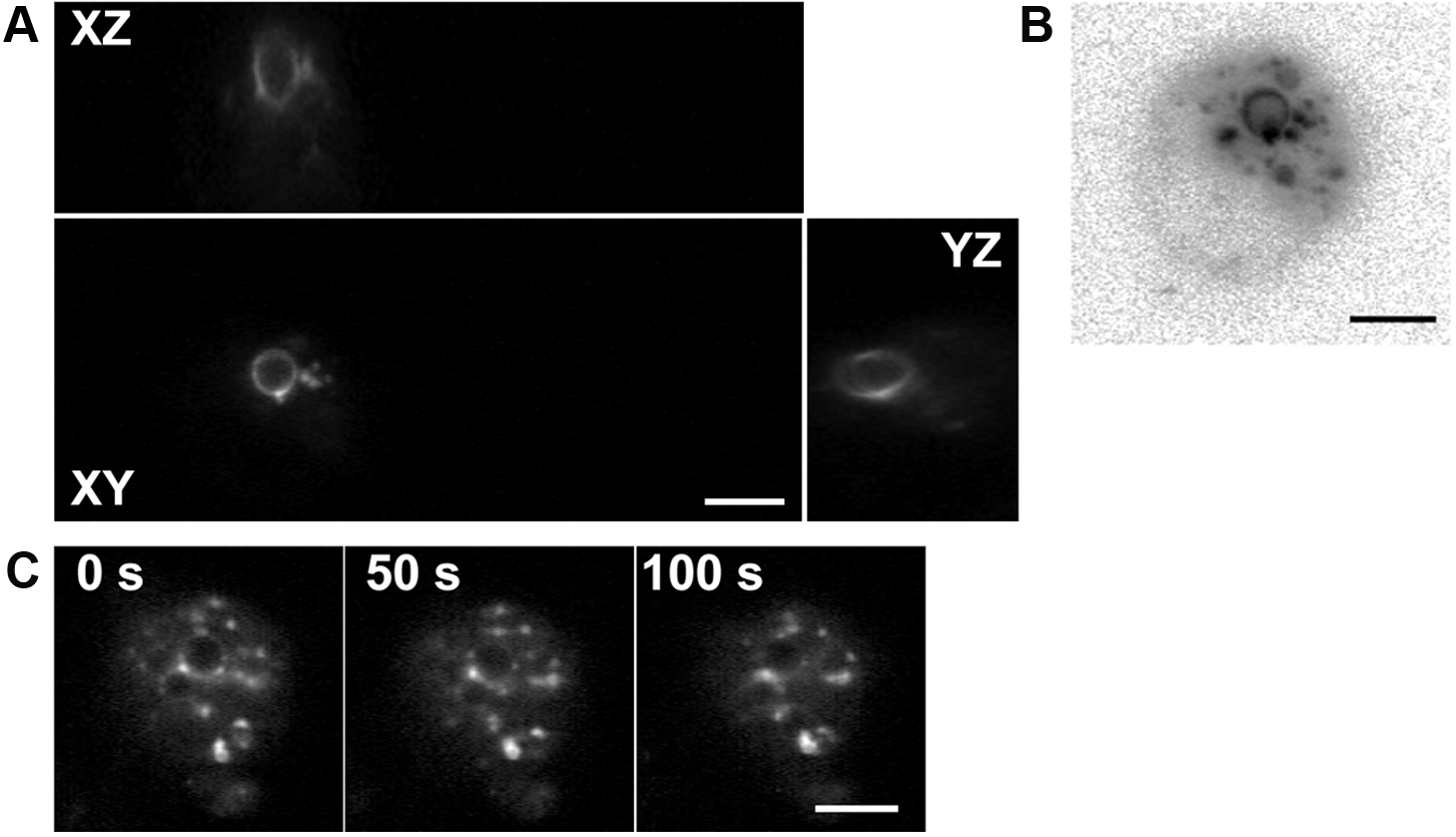

Supplement: Figure S2 — Structure and motility of enlarged vesicles. (A) XY, XZ, and YZ projections of enlarged vesicles in HEK-293 cells transfected with sfGFP-RAB22AI34F. (B) Confocal projection show enlarged RAB22AI34F positive vesicle locating in an intact cell. (C) Time series show mobile activities of buds on the surface of the spherical vesicles. All images were captured using a Zeiss Duoscan confocal microscope with a 63×1.4 NA oil objective, 489 nm laser, 500–550 nm bandpass filter. Scale bar: 10 µm. (TIF) [file pone.0087043.s002.tif]
